# Impulsivity and Emotional Dysregulation in Distress and Self-Perceived Pornography Addiction in Romanian Adults: Advancing the Dysregulation Pathway of “The Pornography Problems Due to Moral Incongruence Model”

**DOI:** 10.1007/s10508-026-03493-3

**Published:** 2026-07-20

**Authors:** Tudor-Daniel Huţul, Andreea Huţul, Adina Karner-Huţuleac, Andrei Corneliu Holman, Joshua B. Grubbs

**Affiliations:** 1 Department of Psychology, Faculty of Psychology and Education Sciences, Alexandru Ioan Cuza University of Iaşi, 3 Toma Cozma Street, 700554 Iaşi, Romania; 2 Department of Communication and Social Research, Sapienza University of Rome, Rome, Italy; 3 Department of Education Sciences, Faculty of Psychology and Education Sciences, Sapienza University of Rome, Iaşi, Romania; 4 Department of Psychology, Center on Alcohol, Substance Use, and Addictions, University of New Mexico, Albuquerque, NM USA

**Keywords:** Problematic pornography use, Impulsivity, Emotional dysregulation, Distress, Self-perceived pornography addiction

## Abstract

“The Pornography Problems Due to Moral Incongruence (PPMI)” is a popular theoretical model addressing negative outcomes of pornography use, through two pathways: (1) the dysregulation pathway and (2) the moral incongruence pathway. The morality pathway has been studied extensively, though the dysregulation pathway has remained underdeveloped. The current study aimed to further investigate the dysregulation pathway from the PPMI model by examining impulsivity and emotional dysregulation. This research was conducted on a sample of 1620 Romanian participants (69.1% females), aged between 18 and 74 years (M = 26.87; SD = 9.15). They completed questionnaires designed to assess dimensions of impulsivity (e.g., “negative urgency,” “positive urgency,” “sensation seeking,” “lack of premeditation,” and “lack of perseverance”), emotional dysregulation, distress, and self-perceived addiction to pornography. Findings revealed that impulsivity and emotional dysregulation relate to distress and self-perceived addiction to pornography, and that these constructs can be integrated into the dysregulation pathway of the PPMI model. This work contributes to the development of the PPMI framework, as well as to a deeper comprehension of the negative outcomes of pornography consumption.

## Introduction

The “Pornography Problems Due to Moral Incongruence” model (PPMI; Grubbs et al., 2019a) is a widely cited theoretical framework for understanding problematic pornography use (Borgogna et al., 2024). The PPMI model postulates that pornography-related problems may originate from two distinct pathways: (1) “pornography problems due to dysregulation,” and (2) “pornography problems due to moral incongruence” (Grubbs et al., 2019a). This theoretical framework has substantially advanced the literature by focusing on moral cognitions and morality-related constructs. However, factors associated with dysregulation pathways have received comparatively limited attention (Lewczuk et al., 2020). These factors have been noted in the PPMI model under the label of “individual differences,” with examples such as coping deficits, impulsivity or emotional dysregulation (Grubbs et al., 2019a). Although there is evidence in the literature suggesting that such individual differences play a key role in problematic pornography consumption (Borgogna & Aita, 2019; Grubbs et al., 2015a; Kraus et al., 2020), the dysregulation pathway remains underdeveloped and requires further exploration (Borgogna et al., 2024; Lewczuk et al., 2020). Thus, to advance the model, it is necessary to test how individual differences contribute to experiencing distress, both directly and indirectly, regarding self-perceived pornography-related problems (Brand et al., 2019a). Importantly, the PPMI model represents an integrative framework that explicitly differentiates between the dysregulation pathway and the morality pathway. In the present work, we therefore address a gap in the PPMI model by examining impulsivity and emotional dysregulation—two extensively studied constructs—within the dysregulation pathway of the PPMI framework. Accordingly, emotional dysregulation and impulsivity are examined as theoretically coherent contributors to the dysregulation pathway rather than being combined with morality-related constructs within the broader PPMI model.

### Impulsivity and Pornography

Impulsivity is a cluster of traits that often result in sudden or rash behavior without premeditation, frequently leading to a dysfunctional outcome (Wetterneck et al., 2012). One of the most influential theoretical frameworks regarding impulsivity is provided by Whiteside and Lynam (2001), who describe impulsivity through four core dimensions: (I) sensation seeking—openness to potentially dangerous or highly stimulating experiences, (II) negative urgency—the tendency to engage in impulsive behaviors aimed at reducing negative emotions, despite these consequences being potentially maladaptive in the long term, (III) lack of perseverance—difficulty maintaining focus on tasks perceived as boring or in completing activities in the presence of distracting stimuli, and (IV) lack of premeditation—acting without considering potential consequences. In addition to the original four dimensions, a fifth dimension, termed (V) positive urgency, was later added (Billieux et al., 2012; Lynam et al., 2006). This dimension refers to the tendency to act impulsively when experiencing positive emotional intensity (Bőthe et al., 2019). Given the multidimensional conceptualization of impulsivity within the UPPS-P model (Billieux et al., 2012; Lynam et al., 2006; Whiteside & Lynam, 2001), it cannot be reduced to a single-dimensional approach. Therefore, general recommendations emphasize that the nature of impulsivity should be addressed using subscales rather than the mean score of the scale.

Several studies have consistently demonstrated that impulsivity represents a vulnerability factor for a broad range of addictive and maladaptive behaviors (Brand et al., 2014; Lee et al., 2019; Verdejo-García et al., 2008), including substance-related and behavioral addictions (Anestis et al., 2007; Billieux et al., 2012; Burnay et al., 2015; Zsila et al., 2018). This also applies in the context of pornography, with which impulsivity has been linked (Testa et al., 2024).

Although individual studies have reported variability in the strength or direction of associations between impulsivity and pornography use—for example, a negative association between impulsivity and frequency of pornography use among men but not women (Carroll et al., 2008), with broader research showing that men are more likely to report self-perceived pornography addiction (Grubbs et al., 2019b), and that pornography use is more strongly associated with adverse psychological and sexual outcomes among men than women (Wright & Tokunaga, 2025), or that impulsivity explains only a small proportion of variance in the frequency of visiting and downloading from pornographic websites (Buzzell et al., 2006)—recent meta-analytic evidence indicates that these associations are overall statistically significant and robust across studies (Akbari et al., 2026). These cumulative findings align with earlier work showing that impulsivity is weakly to moderately associated with pornography use (Beyens et al., 2015; Carroll et al., 2008; Peter & Valkenburg, 2010; Reid et al., 2011).

Building on these results from previous literature, research has investigated how impulsivity is related to problematic pornography use. The findings show that impulsivity positively predicts problematic pornography consumption and may account for up to approximately 15% of the variance in pornography use (Bőthe et al., 2019), a level of variance that aligns with typical ranges reported in the media psychology literature (Valkenburg et al., 2016). Among all the subdimensions of impulsivity, sensation seeking is certainly the most researched dimension in relation to pornography use (Bőthe et al., 2019), a pattern also supported by recent meta-analytic evidence on this association (Wright et al., 2025). Sensation seeking has been consistently linked to both the frequency of pornography use and an increased desire to consume pornographic content (Beyens et al., 2015; Paul, 2009; Peter & Valkenburg, 2010), with sexually compulsive individuals scoring higher on this dimension compared to non-compulsive individuals (Cooper et al., 2000). All these previous findings indicate that individuals with high scores in sensation seeking may use pornography for extended periods and, in certain cases, may develop problematic patterns of usage (Bőthe et al., 2019). At the same time, because sensation seeking is driven by the pursuit of pleasure and excitement, such use may be associated with lower perceived psychological burden, thereby attenuating distress while increasing awareness of intensive use (Bőthe et al., 2019). In such cases, self-perceived addiction may emerge not solely from usage patterns themselves but from the evaluation of these patterns in light of personal, social, or moral standards—a process directly aligned with theoretical formulations suggesting that individual differences contribute to dysregulated pornography use, while the perception of this dysregulation is shaped by personal and sociocultural standards (Wright, 2019).

Negative urgency, or the tendency of individuals to engage in impulsive actions when experiencing intense negative emotions (Cyders & Smith, 2008), is linked to various risky or maladaptive behaviors used to regulate negative affect (Garner et al., 2022). Within these behaviors mentioned in the literature are unprotected sex, one-night stands, transactional sex, sexual aggression perpetration, sex with high-risk individuals (Curry et al., 2018; Cyr et al., 2018; Deckman & DeWall, 2011; Mouilso & Calhoun, 2012). In contrast, these associations appear less applicable to online behaviors such as pornography use. Accordingly, individuals high in negative urgency may be less likely to experience pornography-related distress or self-perceived addiction, a possibility that remains largely unexamined in the existing literature.

In contrast to negative urgency, lack of perseverance has been linked both to various problematic behaviors, such as alcohol consumption (Coskunpinar et al., 2013), cocaine addiction (Verdejo-García et al., 2007), or binge eating (VanderBroek-Stice et al., 2017), as well as to problematic pornography use (Rømer Thomsen et al., 2018). Rømer Thomsen et al. (2018) were the first to link lack of perseverance to problematic pornography use, suggesting that underlying cognitive processes associated with non-substance addictive behaviors may also be relevant in this context. Individuals high in lack of perseverance tend to seek immediate forms of stimulation or relief when confronted with stress or dissatisfaction, a pattern also observed in other addictive behaviors (Canale et al., 2016). When facing distress, such individuals may therefore rely on rapidly accessible coping strategies (Algorani & Gupta, 2024; Folkman & Moskowitz, 2004; Huţul et al., 2024), with pornography representing one of the most readily available options due to its immediacy and ease of access (Borgogna et al., 2022; Bőthe et al., 2021; Lewczuk et al., 2020). Although pornography may function as an adaptive coping strategy in the short term, its use as a primary means of managing distress is unlikely to support healthy emotion regulation and may, over time, exacerbate negative emotional states (Brand et al., 2016, 2019b; Levin et al., 2019). Lack of perseverance has also been associated with a broader pattern of risky behaviors aimed at obtaining rapid relief (Wéry et al., 2018), suggesting a tendency toward short-term solutions in emotionally challenging situations. In this context, individuals high in lack of perseverance may turn to pornography as an immediately accessible coping mechanism, thereby increasing the likelihood of pornography-related distress. At the same time, their focus on rapid relief may limit reflective self-evaluation, making self-perceived addiction less salient even in the presence of frequent use.

In turn, lack of premeditation has not yet been linked to pornography consumption (Bőthe et al., 2019). However, considering its nature (e.g., the tendency of individuals to act without considering the potential consequences of their actions), it is plausible that it could contribute to the distress resulting from pornography use. More specifically, since individuals may resort to pornography as a short-term coping mechanism to manage their negative outcomes (Borgogna et al., 2022; Bőthe et al., 2021; Lewczuk et al., 2020), they may not recognize in those moments that this strategy could be maladaptive in the long run. Thus, over time, pornography use might induce psychological distress in individuals with high scores on lack of premeditation, as well as self-perceived addiction to pornography.

Positive urgency refers to the tendency to act impulsively in response to intense positive emotions (Cyders & Smith, 2008). Although this facet of impulsivity has not been directly examined in relation to pornography use, it is unlikely to be associated with pornography-related psychological distress, which is typically linked to negative affect. In contrast, positive urgency may be related to self-perceived addiction to pornography, as impulsive reactions driven by positive emotional states may influence individuals’ evaluations of their own behavior, increasing the likelihood of labeling their use as problematic even in the absence of marked distress.

Although social and contextual factors may also contribute to pornography-related outcomes, impulsivity remains a relevant individual difference, particularly in the absence of adaptive coping strategies. Prior research indicates that higher impulsivity is associated with greater difficulties in emotion regulation (Schreiber et al., 2012), suggesting that more impulsive individuals may engage in pornography use more frequently. Over time, increased use may heighten the likelihood of experiencing pornography-related distress or self-perceived addiction.

### Emotional Dysregulation and Pornography

Emotion regulation refers to the processes through which individuals monitor, evaluate, and modify their emotional reactions in line with personal goals and situational demands (Berking & Wupperman, 2012). This perspective on emotional regulation involves flexible regulation of an emotional response that an individual employs to cope (Gratz & Roemer, 2004). Hence, it is not surprising that difficulties in emotion regulation are often associated with maladaptive coping strategies in dealing with life distress and are generally recognized in the development of psychopathology (Aldao et al., 2016; Victor & Klonsky, 2016; Visted et al., 2018).

Emotional dysregulation, characterized by inflexible or maladaptive emotion regulation strategies (Coifman & Summers, 2019; D’Agostino et al., 2017), has been linked to problematic pornography use (Walton et al., 2017). In this context, pornography may function as an easily accessible coping behavior aimed at alleviating negative emotions, thoughts, or urges (Ross et al., 2012; Wéry et al., 2019), particularly when emotional states are intense and difficult to manage (Algorani & Gupta, 2024; Folkman & Moskowitz, 2004; Huţul et al., 2024). However, reliance on pornography as a primary coping mechanism may reflect dysfunctional emotion regulation, particularly when it replaces more adaptive strategies, thereby increasing vulnerability to negative psychological outcomes (Borgogna et al., 2022; Bőthe et al., 2021; Grubbs et al., 2019c).

From the perspective of the PPMI framework, difficulties in emotion regulation may contribute to negative pornography-related outcomes, as the short-term alleviating effects of pornography use can reinforce its adoption as a compensatory coping strategy (Büsche et al., 2022; Qu et al., 2024), ultimately increasing the likelihood of distress and self-perceived addiction (Grubbs et al., 2019a). Accordingly, examining difficulties in emotion regulation as part of the dysregulation pathway is imperative.

### The Role of Self-Perceived Pornography-Related Problems and Distress

Self-perceived pornography addiction refers to an individual's conviction that they are unable to regulate their use of pornography and that their difficulties in regulation are an addiction (Karner-Huţuleac & Huţul, 2023; Lewczuk et al., 2020). This construct is often measured in the literature through brief measures (Lewczuk et al., 2020), such as “I am addicted to internet pornography” (Grubbs et al., 2019b), though longer measures also exist (e.g., the Problematic Pornography Consumption Scale; Bőthe et al., 2018; the Cyber Pornography Use Inventory 9; Grubbs et al., 2010; the Brief Pornography Screen; Kraus et al., 2020). Numerous studies provide evidence that some individuals experience self-perceived addiction to pornography (Grubbs et al., 2015a, 2018a). Additionally, self-perceived addiction to pornography comprises three main components: (1) individuals' evaluations regarding their internet pornography use, such as perceived dysregulation or compulsivity; (2) perceptions related to individuals' behavioral engagement regarding pornography use, such as perceived disruptions or efforts expended for access; and (3) psychological distress regarding an individual's internet pornography use, such as experienced emotional distress (Grubbs & Perry, 2019).

Self-perceived addiction to pornography has been consistently associated with psychological distress and other negative outcomes (Dover et al., 2024; Grubbs et al., 2015c, 2019a), with impacts extending from the individual to broader social functioning (Alves & Cavalhieri, 2020; Kingston et al., 2009; Love et al., 2015; Perry, 2017). Individuals may come to label their pornography use as addictive for multiple reasons, including moral, social, and relational concerns, as well as behavioral factors such as frequency, duration, or use in socially inappropriate contexts (Twohig & Crosby, 2010). In addition to these factors, emotional dysregulation has been identified as an important contributor to self-perceived addiction to pornography (Lew-Starowicz et al., 2020; Walton et al., 2017). Thus, when individuals with self-perceived addiction to pornography use pornography, it is recognized as a maladaptive coping strategy but also as a form of experiential avoidance, which is an additional effort to cope with and manage thoughts and negative feelings, despite the fact that this strategy can lead to additional negative outcomes (Wetterneck et al., 2012). Among the negative outcomes of self-perceived addiction to pornography use, we find in the literature associations with greater levels of distress both simultaneously (Grubbs et al., 2015a, 2015b) and over time (Grubbs et al., 2015b), higher levels of alcohol consumption (Morelli et al., 2017), problematic gaming (Bőthe et al., 2015), higher levels of relational anxiety (Leonhardt et al., 2018), difficulties and complications in one's religiosity and spiritual life (Grubbs et al., 2017; Wilt et al., 2016), or lower levels of sexual satisfaction or well-being (Blais-Lecours et al., 2016; Vaillancourt-Morel et al., 2017).

Also, previous literature suggests a link between distress and pornography consumption (Perry, 2018; Privara & Bob, 2023). The studies indicating that distress is associated with pornography use are extremely numerous (Gola & Potenza, 2016; Grubbs & Kraus, 2021; Grubbs et al., 2015b, 2018b, 2019a; Huţul & Karner-Huţuleac, 2023a; Kraus et al., 2015; Seyedzadeh Dalooyi et al., 2023; Tan et al., 2022; Twohig et al., 2009).

In this regard, studying factors that contribute to distress and self-perceived addiction to pornography, such as impulsivity or emotional dysregulation, represents a step forward both for theoretical knowledge and for practice. Psychotherapists and mental health workers can thus base their intervention when encountering these outcomes resulting from pornography use in their clinical practice. Moreover, within the original formulation of the PPMI model, the dysregulation and moral incongruence pathways are conceptualized as distinct routes that may coexist but are not necessarily dependent on one another (Grubbs et al., 2019a). The present work focuses exclusively on the dysregulation pathway and does not test interactions or moderating effects involving morality-related constructs, as the primary aim is to more comprehensively understand the role of individual differences within the dysregulation pathway. Accordingly, moral disapproval (or moral incongruence indicators) is not modeled here as a moderator of dysregulation-related distress.

### The Present Study

Based on previous literature, the current study aims to advance knowledge by testing impulsivity and emotional dysregulation within the dysregulation pathway of “The Pornography Problems Due to Moral Incongruence” model. To achieve our objective, we investigated the relationships between impulsivity, emotional dysregulation, distress, and self-perceived addiction to pornography. Additionally, we utilized a prediction model for distress and self-perceived addiction to pornography. Although the original formulation of the PPMI model conceptualizes self-perceived addiction to pornography as a proximal construct linking antecedents to psychological distress, the present study does not aim to test the model in its entirety. Rather than specifying the full mediational sequence proposed by the PPMI, self-perceived addiction to pornography and distress are examined in parallel rather than as components of the full PPMI causal chain.

The novelty of our research lies in (1) exploring impulsivity and emotional dysregulation within “the Pornography Problems Due to Moral Incongruence” model and (2) exploring these constructs in an understudied context, such as Romania, where the literature on pornography consumption is nearly nonexistent. Examining these processes in Romania is particularly relevant, given the sociocultural context in which topics such as sexuality and pornography use remain strongly stigmatized and considered taboo (Gergely & Rusu, 2023; Huţul et al., 2025a). This situation stems from historical restrictions on sexual education during the communist period, combined with the ongoing influence of conservative religious norms associated with Orthodox Christianity, which have contributed to a limited public discourse surrounding sexuality and related behaviors (Andrei & Branda, 2015; Huţul et al., 2025a, 2025b; Pop-Elecheş, 2010; Turcescu & Stan, 2005). This context suggests that psychological dysregulation processes may play a more salient role in pornography-related distress and self-perceived addiction, potentially shaping these outcomes differently than in sociocultural contexts where sexuality is less morally regulated.

#### Hypotheses

##### H1

Negative urgency, positive urgency, lack of premeditation, sensation seeking, and lack of perseverance will be positively associated with both general emotional distress and self-perceived addiction to pornography specifically.

##### H2

Emotional dysregulation will be positively associated with distress and self-perceived addiction to pornography.

**Fig. 1:**
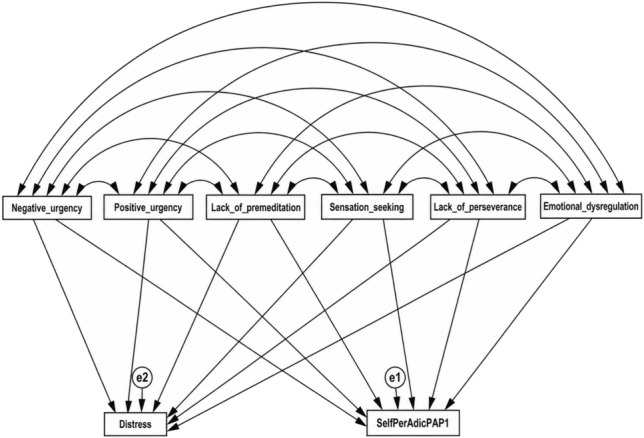
The proposed model

The proposed model is shown in Fig. 1.

## Method

### Participants and Procedure

**Fig. 2:**
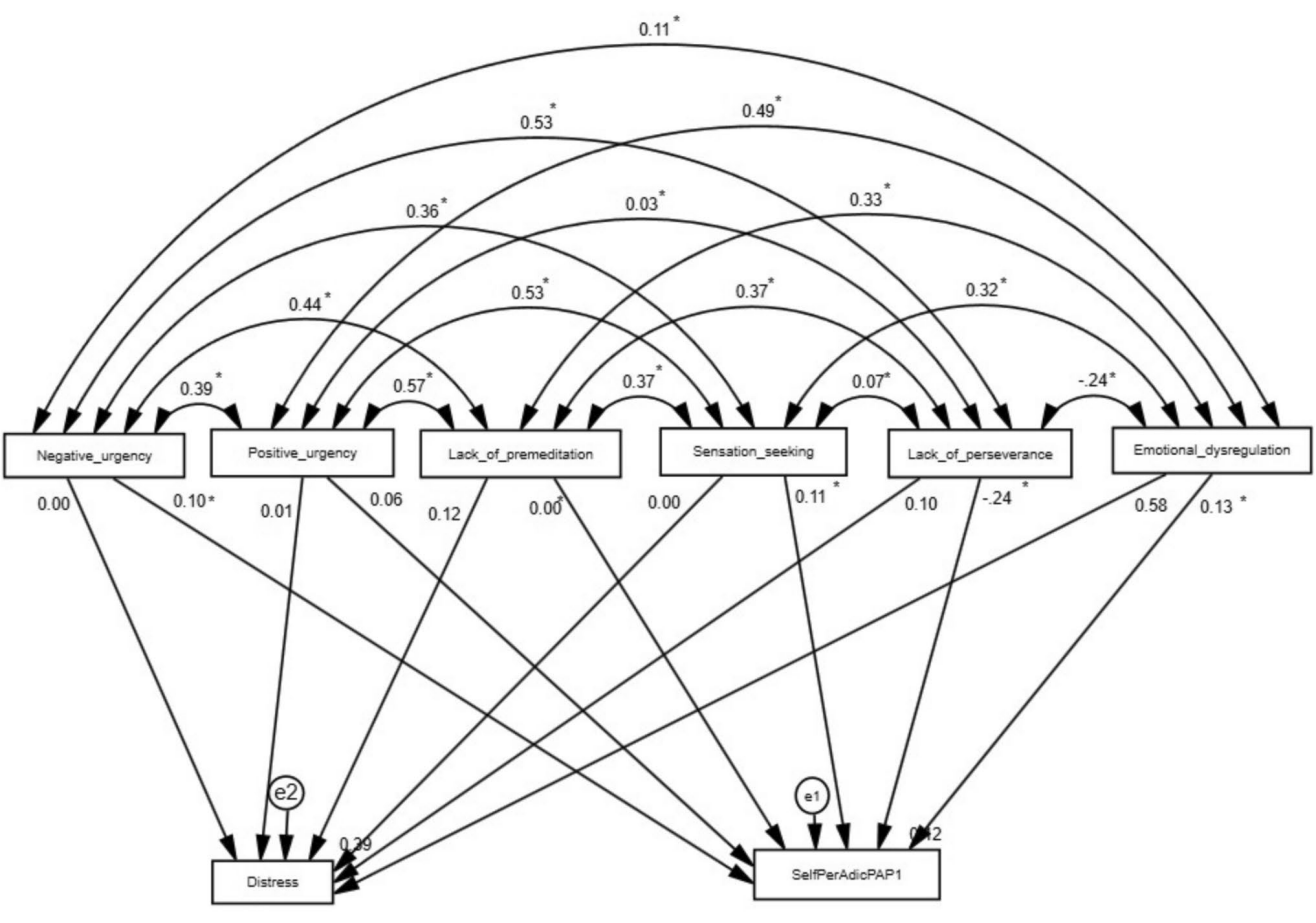
Path analysis of the predictors of distress and self-perceived addiction to pornography. Standardized path coefficients are reported. *p < .001. SelfPerAdicPAP1 = Self-Perceived Addiction to Pornography

An a priori power analysis was conducted using G*Power 3.1 (Faul et al., 2007) for multiple regression models, approximating the most complex endogenous regression in the proposed SEM. With six predictors, a medium effect size (*f*^*2*^ = 0.15),* α* = .05, and desired power of .95, the required sample size was N = 146. Because the SEM includes two endogenous variables, the analysis was based on the equation with the largest number of predictors; both endogenous variables had the same number of predictors, therefore the required N was identical (Fig. 2).

In this study, 1620 individuals aged 18–74 years (M = 26.87; SD = 9.15) participated. Among the total participants, the majority were female (69.1%), from an urban area (57%), with bachelor's degrees (43.2%), and in a relationship (54%), with an average duration of the relationship of 45.54 months. The participants’ characteristics are detailed in Table 1.

**Table 1:** Sample characteristics

		N	%
Sex	Male	500	30.9
	Female	1120	69.1
	Non-binary	0	0
Place of origin	Rural	696	43
	Urban	924	57
Level of education	High school diploma	608	37.6
	Bachelor’s degree	700	43.2
	Master’s degree	289	17.8
	Doctorate’s degree	23	1.4
Marital status	In a relationship (not married)	768	47.4
	Married	385	23.8
	Single	467	28.8
		M	SD
	Age (in years)	26.87	9.15
	Duration of the relationship (in months)	45.54	75.91

The present research employed snowballing technique and convenience sampling. To assess the surveys, a battery of instruments was created on the freely available online platform Google Forms. In an attempt to achieve a balanced representation from the population of Romania, the questionnaire link was disseminated on social media platforms such as Facebook, Reddit, and Telegram in groups representing the most important cities in Romania (e.g., groups for people living in Bucharest, Iaşi, Cluj, Braşov, or Timişoara). The selected groups for distributing the link were extremely general (e.g., “Locuitori din Braşov” [“People from Braşov”] or “Grupul cartierului Tătăraşi din Iaşi” [“Residents of the Tătăraşi neighborhood in Iaşi”]). We chose this approach to try to avoid specific background groups such as student groups from various universities (e.g., “Grupul studenţilor din România” [“Romanian Students’ Group”] or “Grupul studenţilor de la psihologie” [“Psychology Students’ Group”]) and to obtain responses from individuals with a variety of professional and social backgrounds. The utilization of this approach for sharing the link to the battery of instruments has been previously employed in literature to attempt to provide a more comprehensive insight into the subject of pornography (Huţul & Karner-Huţuleac, 2024b; Huţul et al., 2025b). The advertisement posted on the specified social media groups stated that the present research is conducted as a university study on psychological factors related to the use of pornography, that participation is entirely voluntary and that participants will not receive compensation for taking part, and that it is open only to adults aged 18 and above.

Prior to completing our survey, volunteers were asked to carefully read the informed consent. Within it, they were informed that participation was entirely voluntary and they would not be compensated in any way for their participation, that they could request assistance for any questions they had regarding the research, that all information would be kept confidential. Additionally, the contact phone number and email address of the corresponding author of this study were provided in the survey description. The average completion time for the survey battery was approximately 10–12 min.

The data collection period took place between October 1, 2023 and August 14, 2024. Before constructing the final database, the first four authors of the present work conducted a manual verification process of the responses in order to identify possible automated answers generated by a bot or other software, or to identify “mischievous respondents,” as indicated in the literature (Lawrence et al., 2023). Also, a search was conducted for possible responses submitted in an unusually short amount of time, such as answers provided in less than 5–6 min. This approach was also used in other studies involving the population from Romania (Ciobanu et al., 2025), being in line with recommendations in the literature (Golds, 2023). Moreover, our battery of instruments included 3 attention-check items (Thomas & Clifford, 2017). In total, our survey received 1683 responses. Of these, 32 were removed as “mischievous respondents” or excessively fast responses, and 43 failed the control questions (12 eliminated responses had both mischievous indicators or overly fast responses and failed the control questions). Thus, in total, 63 responses were removed, and from the initial 1683 responses, the final database consisted of 1620 responses.

Regarding the eligibility criteria for participation in this study, we included the legal age in Romania of 18 years, reached at the moment when volunteers complete the questionnaire.

### Measures

All measures were translated and culturally adapted into Romanian using a standardized back-translation procedure involving independent forward translations, expert reconciliation, and back-translation. Recommendations for translation and adaptation of the scales were made according to protocol (Maneesriwongul & Dixon, 2004; Sousa & Rojjanasrirat, 2011). Two separate teams, comprising psychologists experienced in the research of variables and constructs within the sphere of sexuality and pornography, along with professional translators, independently translated each instrument into two versions. Subsequently, a comprehensive comparison and discussion were conducted to create a consolidated translation for each scale in Romanian. These translations were then back-translated into English by qualified professionals. Any disparities between the back-translated versions and the original measures were identified and resolved through discussion, culminating in the final versions of each scale in Romanian. The back-translation method ensured the retention of the conceptual meaning of the original measures. This approach to translating instruments from English into Romanian has previously been employed in other studies that have addressed the topic of pornography use (Huţul & Karner-Huţuleac, 2025; Pricope et al., 2026).

Regarding the questions related to pornography, participants were provided with the following description in accordance with the guidelines from the literature (Kohut et al., 2020), a definition also used in other studies (Grubbs et al., 2022), including in Romania (Huţul & Karner-Huţuleac, 2023b; Huţul et al., 2024): “Pornography refers to any sexually explicit films, video clips, or pictures displaying the genital area, with the intention to sexually arouse the viewer; this may be seen on the internet, in a magazine, in a book, or on television.”

*Impulsivity.* To assess individuals’ level of impulsivity, we utilized The Short Impulsive Behavior Scale (SUPPS-P; Cyders et al., 2014; Zsila et al., 2020), developed by Billieux et al. (2012) from the original 59-item Impulsive Behavior Scale (UPPS-P; Lynam et al., 2006). This scale consists of 20 items grouped into 5 impulsivity subscales, with 4 items each dimension, as follows: (I) “negative urgency” (e.g., “I often make matters worse because I act without thinking when I am upset,”) (II) “positive urgency” (e.g., “I tend to act without thinking when I am really excited”) (III) “sensation seeking” (e.g., “I welcome new and exciting experiences and sensations, even if they are a little frightening and unconventional”) (IV) “lack of premeditation” (e.g., “Before making up my mind, I consider all the advantages and disadvantages”) and (V) “lack of perseverance” (e.g., “I am a productive person who always gets the job done”) All items of the instrument are scored on a four-point Likert scale, ranging from 1 = “I agree strongly” to 4 = “I disagree strongly.” The higher scores indicated a higher level of each subscale. We examined the validity of this scale in the Romanian population through a confirmatory factor analysis (CFA) in AMOS 22, and we found that some of the model fit indices were outside the recommended intervals: *χ*^*2*^(164) = 1262.54, *p* < .001, CFI = .90 < .95, TLI = .88 < .90, while others suggested a good fit: RMSEA = .06 < .08, SRMR = .07 < .08. We found one item in the lack of perseverance subscale (i.e., item 7) that had a low standardized regression weight in relation to its factor (.19) and a low squared multiple correlation (.04), and we eliminated this item. Moreover, the modification indices suggested that other important sources of discrepancy between the measurement model and the data are the high associations between the error variances of seven pairs of items, and we respecified the model with these associations. The CFA on the final model that included these modifications indicated an adequate model fit: *χ*^*2*^(139) = 699.66, *p* < .001, CFI = .95, TLI = .93, RMSEA = .05, SRMR = .05. The Alpha Cronbach coefficients are = .75 for “Negative urgency”, = .80 for “Positive urgency”, = .72 for “Sensation seeking”, = .76 for “Lack of premeditation”, and = .67 for “Lack of perseverance”.

*Emotional dysregulation*. To assess individuals' level of emotional dysregulation, we utilized the Difficulties in Emotion Regulation Scale (DERS; Gratz & Roemer, 2004). This scale comprises 36 self-report items, rated on a 5-point Likert scale from 1 = “almost never” to 5 = “almost always,” regarding individuals' relationship with their emotions. The instrument includes 6 subscales: (I) “nonacceptance of emotional responses” (e.g., “When I’m upset, I become angry with myself for feeling that way”) (II) “difficulty engaging in goal-directed behavior” (e.g., “When I’m upset, I have difficulty concentrating”) (III) “impulse control difficulties” (e.g., “When I’m upset, I have difficulty controlling my behaviours,”) (IV) “lack of emotional awareness” (e.g., “When I’m upset I take time to figure out what I’m really feeling”) (V) “limited access to emotion regulation strategies” (e.g., “When I’m upset, I believe that wallowing in it is all I can do”) and (VI) “lack of emotional clarity” (e.g., “I have no idea how I am feeling”) This scale has demonstrated its psychometric properties over time (Hallion et al., 2018), including in studies involving the Romanian population (Antoniac et al., 2024; Bostan & Zaharia, 2016; Matei-Mitacu et al., 2024). Higher scores indicated a higher level of emotional dysregulation. The results of the CFA in AMOS 22 on this scale indicated a poor model fit: *χ*^*2*^(588) = 6723.54, *p* < .001, CFI = .83, TLI = .81, RMSEA = .08, SRMR = .1. We found four items, i.e., two in the Lack of Emotional Awareness subscale (Items 17 and 34), one in the Impulse Control Difficulties subscale (Item 24) and one in the Limited Access to Emotion Regulation Strategies subscale (item 22) with low standardized regression weights in relation to their factors and low squared multiple correlations, and we eliminated these items. We also found high associations between the error variances of eleven pairs of items. The CFA on the final model that included these modifications indicated an adequate model fit: *χ*^*2*^(415) = 2208.41, *p* < .001, CFI = .95, TLI = .94, RMSEA = .05, SRMR = .04. The scale reliability was excellent (Cronbach’s α = .94).

*Distress*. To measure individuals' distress, we employed an adapted version of the Kessler Psychological Distress Scale (K-6; Kessler et al., 2003), tailored to pornography use, in accordance with recommendations from the literature for addressing this scale (which represents a global measure for distress) in the context of pornography use (Huţul & Karner-Huţuleac, 2024b). The adapted scale for psychological distress resulting from pornography consumption comprises 6 items and 6 specific recommendations addressed to participants (e.g., “During the last 30 days, about how often did you feel hopeless?” with the recommendation: “Participants were required to reflect on the frequency with which they experienced hopelessness as a result of their consumption of pornography”). The instrument comprises 6 items on a 5-point Likert scale, ranging from 0 = “none of the time” to 4 = “all of the time.” Although this adapted scale has been previously used in the context of pornography use (Tan et al., 2022), including on the Romanian population (Huţul & Karner-Huţuleac, 2024b; Petroiu et al., 2025), we examined its factorial validity through an exploratory factor analysis with principal axis factoring in SPSS 24.0. The Kaiser–Meyer–Olkin (KMO) measure of sampling adequacy (0.869) and the Bartlett’s test (*p* < 0.001) indicated the adequacy of the data for factor analysis. Results indicated one factor with an Eigenvalue above 1 (3.83), accounting for 63.81% of the data variance. The Parallel Analysis also indicated one factor with an eigenvalue higher than its corresponding 95th percentile eigenvalue derived from random data, suggesting the appropriateness of the unifactorial solution (Glorfeld, 1995). The factor loadings of the items ranged from .62 to .87, and the Cronbach’s alpha of this one-factor scale (0.88) indicated excellent reliability. Higher scores indicate an elevated level of psychological distress resulting from pornography use. The scale reliability was excellent (Cronbach’s α = 0.88).

*Self-perceived addiction to pornography*. Measuring this construct involved a single item (e.g., “I am addicted to internet pornography”), in accordance with previous literature (Grubbs et al., 2018c; Lewczuk et al., 2020), which has also employed this item in studies conducted with Romanian populations (Huţul & Karner-Huţuleac, 2025; Petroiu et al., 2025). This item is derived from the Cyber-Pornography Use Inventory (CPUI-9; Grubbs et al., 2010), and is rated on a 7-point Likert scale ranging from 1 = “strongly disagree” to 7 = “strongly agree.” Higher scores indicate a high level of self-perceived addiction to pornography.

*Sociodemographic data.* Participants provided the following information: age, sex, level of education, relationship status, relationship duration, and place of origin.

### Statistical Analysis

We verified the normality of data distribution and the association between the main concepts of the research. Statistical analyses were performed using the SPSS program, version 26 (George & Mallery, 2019).

Then, to evaluate Dysregulation pathway from PPMI model, we conducted a path analysis, using IBM AMOS 22 (Arbuckle, 2024). Following the standards adopted in the literature (Hu & Bentler, 1999; Kline & Little, 2023), goodness of fit was assessed using the following criteria: CFI, and AGFI values greater than .90, GFI and TLI values greater than .95, RMSEA value lower than .08 (with an associated p > .05), and SRMR value lower than .08.

Finally, we examined the moderating effects of gender on the structural relationships in our model using multi-group analyses, in light of the sex variations in the consumption and mental health effects of pornography observed by past research (Grubbs et al., 2019b; Wright & Tokunaga, 2025).

## Results

### Preliminary Data Analyses and the Associations Among the Main Variables

We computed the Skewness and Kurtosis values to assess the normality of the distributions (Table 2). All the Skewness values were within the 2/-2 limit and Kurtosis values were within 7/-7, indicating normality, as suggested by Hair et al. (2010) and Byrne (2013).

**Table 2:** Descriptive statistics and associations among the main variables

	M (SD)	Skewness (SE)	Kurtosis (SE)	1	2	3	4	5	6	7	8
1. Impulsivity – negative urgency	11.23 (2.01)	.01 (.06)	.20 (.12)	–							
2. Impulsivity – positive urgency	9.79 (2.35)	.46 (.06)	− .27 (.12)	.38*	–						
3. Impulsivity – lack of premeditation	11.26 (1.9)	.21 (.06)	.25 (.12)	.43*	.56*	–					
4. Impulsivity – lack of perseverance	11.26 (2.1)	.02 (.06)	− .19 (.12)	.46*	.28*	.42*	–				
5. Impulsivity – sensation seeking	9.47 (2.88)	.16 (.06)	− .58 (.12)	.36*	.53*	.37*	.45*	–			
6. Emotional dysregulation	89.63 (24.05)	.19 (.06)	− .48 (.12)	.09*	.49*	.33*	.02	.31*	–		
7. Distress	17.89 (6.83)	.47 (.06)	− .42 (.12)	.17*	.36*	.35*	.16*	.24*	.60*	–	
8. Self-perceived addiction to pornography	1.73 (1.34)	1.96 (.06)	3.2 (.12)	.04	.21*	.06*	.01	.20*	.25*	.16*	–

^*^p < .001

### Path Analysis

Results of the path analysis for Dysregulation Pathway from PPMI model indicated good fit values. χ^2^(1) = 1.56; p = .21; CFI = 1; GFI = 1; AGFI = .99; TLI = .99; RMSEA = .001; upper CI = .07; p = .77; SRMR = .002.

Emotional dysregulation was strongly and positively associated with distress (*β* = .58; *p* < .001) and with self-perceived addiction to pornography (*β* = .13; *p* < .001). Negative urgency was positively associated with self-perceived addiction to pornography (*β* = .10; *p* = .002), but was not significantly associated with distress (*β* = .001; *p* = .97). Positive urgency was not significantly associated with distress (*β* = .01; *p* = .79) and with self-perceived addiction to pornography (*β* = .06; *p* = .06). Lack of premeditation was positively associated with distress (*β* = .12; *p* < .001), but was not significantly associated with self-perceived addiction to pornography (*β* = -.01; *p* = .88). Sensation seeking was positively associated with self-perceived addiction to pornography (*β* = .11; *p* < .001), but not with distress (*β* = .003; *p* = .88). Lack of perseverance was positively associated with distress (*β* = .10; *p* < .001) and negatively associated with self-perceived addiction to pornography (*β* = -.24; *p* < .001).

The model explained 39.4% of the variance in distress and 12.3% variance in self-perceived addiction to pornography. Bootstrap analyses with 2000 resamples were conducted to assess the robustness of the parameter estimates.

### Model Comparison

To address concerns that the excellent fit indices might reflect overfitting, we compared the proposed model with a theoretically plausible and more parsimonious alternative model in which impulsivity facets predicted self-perceived addiction to pornography, with distress included as a secondary outcome. Model comparisons were conducted using the Akaike Information Criterion (AIC) and the Bayesian Information Criterion (BIC), with lower values indicating better relative fit. The proposed model showed lower AIC and BIC values (AIC = 71.56; BIC = 260.21) than the alternative model (AIC = 324.92; BIC = 448.89), supporting its selection and suggesting that the excellent fit indices do not reflect overfitting. In addition, we tested a further theoretically plausible alternative model in which emotional dysregulation was specified as an endogenous variable predicted by impulsivity facets, alongside distress and self-perceived addiction to pornography. This alternative specification showed substantially higher information criteria values (AIC = 586.19; BIC = 764.09) compared to the proposed model, further supporting the selection of the proposed model.

### Testing the Moderating Effects of Sex

The multigroup analysis first entailed evaluating the chi-square difference between the initial model, in which the structural path coefficients were freely estimated across sex groups, with a fully constrained model, in which all these coefficients were constrained to be equal in the two sex groups (Table 3). Results indicated that the latter model, χ^2^(14) = 44.75 had a significantly worse fit than the unconstrained one, χ^2^(2) = 8.59, Δχ^2^ = 36.16, Δ*df* = 12, *p* < .001, suggesting that there are structural path coefficients that vary significantly between sex. We investigated these variations by sequentially constraining each coefficient to be equal across sexes and comparing the resulting model to the unconstrained one. The results of these 12 model comparisons are reported in Table 3 (Δ*df* = 1 for all comparisons) and indicated three structural paths that differed significantly between sex. First, the effect of emotional dysregulation on self-perceived addiction to pornography was stronger in men (*β* = .20; *p* < .001) than in women (*β* = .16; *p* < .001). Second, the effect of lack of premeditation on self-perceived addiction to pornography was significant in men (*β* = .14; *p* = .04) but not in women (*β* = − .05; *p* = .22). Similarly, the effect of sensation seeking on distress was significant in men (*β* = .10; *p* = .02) but not in women (*β* = − .02; *p* = .50).

**Table 3:** Comparisons between models with each path constrained and the unconstrained model

Path constrained		χ^2^	χ^2^ difference from the unconstrained model	*p* of the difference from the unconstrained model
Emotional dysregulation to	Distress	8.82	.63	.63
	SPAP	12.46	3.87	.049
Negative urgency to	Distress	10.27	1.68	.20
	SPAP	9.09	.50	.48
Positive urgency to	Distress	10.41	1.81	.18
	SPAP	8.77	.18	.67
Lack of premeditation to	Distress	9.14	.55	.46
	SPAP	14.22	5.62	.02
Sensation seeking to	Distress	14.21	5.62	.02
	SPAP	10.05	1.46	.23
Lack of perseverance to	Distress	8.77	.19	.66
	SPAP	8.63	.04	.84

*SPAP* Self-perceived addiction to pornography

## Discussion

The aim of the present work was to develop Pathway 1, “Pornography Problems Due to Dysregulation,” within the dysregulation pathway of the PPMI framework (Grubbs et al., 2019a), which initially involved only a series of proposed individual differences. Based on the literature, we tested impulsivity subscales and emotional dysregulation within Pathway 1, two of the constructs that have previously been linked with pornography use. Both have shown their significant role in pornography-related problems, contributing to experiencing distress and self-perceived addiction to pornography.

First and foremost, our findings showed that lack of premeditation was significantly and positively associated with distress and self-perceived pornography addiction. Regarding psychological distress related to pornography consumption, the result can be explained by the fact that individuals with high scores on lack of premeditation are more likely to act without considering the long-term consequences of their actions (Billieux et al., 2012; Lynam et al., 2006; Whiteside & Lynam, 2001), which can lead to dysfunctional outcomes (Wetterneck et al., 2012), particularly in moments when stress and emotions are extremely intense. Thus, it is plausible that they use pornography as a short-term coping mechanism to avoid negative states or to quickly obtain a sense of relief (Borgogna et al., 2022; Bőthe et al., 2021; Lewczuk et al., 2020); however, in the long term, pornography may lead to negative consequences and impact the consumer's life, ultimately resulting in psychological distress (Brand et al., 2016, 2019b; Levin et al., 2019). In contrast, when distress occurs, individuals may come to realize that they have acted impulsively and developed a pattern of turning to pornography during impulsive moments, which could contribute to a self-perceived addiction.

Additionally, regarding lack of perseverance, our study has revealed that this dimension of impulsivity is significantly linked with psychological distress related to pornography use, but not with self-perceived addiction to pornography. These results can be explained by the fact that individuals with high scores on lack of perseverance seek rapid and immediate solutions for emotional discomfort (Borgogna et al., 2022; Bőthe et al., 2021; Lewczuk et al., 2020), with pornography being one of the most accessible coping mechanisms (Algorani & Gupta, 2024; Folkman & Moskowitz, 2004; Huţul et al., 2024). However, in the long term, this coping mechanism can exacerbate suffering (Brand et al., 2016, 2019b; Levin et al., 2019). Conversely, because individuals with lack of perseverance are focused on quick solutions, they may not develop a self-perceived addiction to pornography. Also, our findings contribute to the literature on addictive behaviors, which has shown that lack of perseverance is associated with negative behaviors such as risky sexual behavior (Wéry et al., 2018), alcohol consumption (Coskunpinar et al., 2013), cocaine addiction (Verdejo-García et al., 2007), or binge eating (VanderBroek-Stice et al., 2017).

Furthermore, our results suggest that positive urgency showed a trend-level positive association with self-perceived pornography addiction. A potential explanation for this finding may lie in the fact that impulsive tendencies resulting from positive emotions (Cyders & Smith, 2008) can influence individuals’ self-perception regarding their patterns of pornography use. For example, if a person frequently turns to pornography when experiencing positive emotions, they may come to perceive themselves as addicted. Additionally, the fact that this subdimension is associated with positive emotions may explain why positive urgency was not linked with psychological distress related to pornography use, as distress is typically associated with negative emotions.

Regarding sensation seeking, it significantly and positively predicted self-perceived pornography addiction, but not psychological distress related to the use of pornography. The lack of significance between sensation seeking and psychological distress related to pornography use can be explained by the fact that this dimension of impulsivity opens individuals to various experiences and brings enjoyment from exciting activities (Whiteside & Lynam, 2001) as can be the case with pornography use. Additionally, our result is supported by the fact that sensation seeking is linked to the frequency of pornography use and a higher desire to engage in it (Beyens et al., 2015; Paul, 2009; Peter & Valkenburg, 2010). Thus, individuals with higher sensation seeking may perceive pornography use as a pleasurable activity and, in this sense, may not associate it with negative outcomes, such as psychological distress. However, the finding that sensation seeking is linked to self-perceived addiction to pornography can be explained by the fact that individuals, even if they do not experience distress regarding their consumption, may later become aware that they have exceeded certain social or personal norms related to pornography use, which can lead to labeling the behavior as addictive. Thus, this self-perception may arise more from an evaluation of one's own behavior in relation to social or personal expectations rather than from psychological distress related to the use of pornography.

Finally, regarding the subdimensions of impulsivity, the fact that negative urgency is not linked to either distress or self-perceived addiction to pornography can be explained by several factors. One such factor is that negative urgency has been associated with risky sexual behaviors in real life, such as unprotected sex, one-night stands, or sex with high-risk partners (Curry et al., 2018; Cyr et al., 2018; Deckman & DeWall, 2011; Mouilso & Calhoun, 2012). In contrast, pornography use occurs almost exclusively in an online environment. Additionally, another aspect that may explain our findings is related to the fact that individuals with high negative urgency have a heightened tendency towards risky behaviors, which, by their nature, differ from pornography use.

Moreover, our study revealed that emotional dysregulation has been positively associated with distress and self-perceived addiction to pornography. This finding is in line with the literature, which has shown that emotional dysregulation is linked to difficulties in processing emotions and problematic pornography use (Walton et al., 2017). In this context, pornography use serves as a dysfunctional means through which individuals with high emotional dysregulation may attempt to regulate their own negative feelings, thoughts, or sensations (Algorani & Gupta, 2024; Folkman & Moskowitz, 2004). In other words, pornography is used as a coping mechanism to deal with the challenges individuals face (Huţul et al., 2024) in the absence of adaptive emotional regulation and healthy coping strategies (Borgogna et al., 2022; Bőthe et al., 2021; Grubbs et al., 2019c; Lewczuk et al., 2020). Moreover, our results should not surprise us given that emotional dysregulation may play a significant role in the choice to engage in pornography consumption, considering that the use of this content can have compensatory effects that alleviate, at least momentarily, unpleasant feelings (Büsche et al., 2022; Qu et al., 2024). Among these experienced states that can be alleviated and that can lead an individual to escape from pornography, we also find anxiety or stress (Grubbs et al., 2015b; Mehmood Qadri et al., 2023; Privara & Bob, 2023). However, in this way, when individuals with emotional dysregulation turn to pornography to cope with emotional difficulties, a vicious cycle can be created, as once the feelings of relief dissipate, negative outcomes such as distress and self-perceived addiction to pornography can be more strongly experienced.

Taken together, negative urgency, positive urgency, lack of premeditation, sensation seeking, lack of perseverance, and emotional dysregulation explained 39.4% of the variance in distress and 12.3% of the variance in self-perceived addiction to pornography. A potential explanation for these results may lie in the complex role that impulsivity and emotional dysregulation play in determining the distress and self-perceived addiction to pornography experienced by an individual. For instance, impulsivity, often characterized by a tendency to act without thorough consideration and a need for immediate gratification, can lead an individual to behaviors that may be harmful to their emotional state (Lynam et al., 2006; Wetterneck et al., 2012; Whiteside & Lynam, 2001). As for emotional dysregulation, it can amplify the negative effects stemming from impulsivity, consequently leading to an increased risk of experiencing distress and self-perceived addiction to pornography. This can occur as a result of emotional dysregulation often being associated with intense moments of distress and being frequently recognized in the development of psychopathology (Aldao et al., 2016; Victor & Klonsky, 2016; Visted et al., 2018). In this regard, it is important to mention that emotional dysregulation is one of the factors in the literature that has been noted to influence self-perceived addiction to pornography (Lew-Starowicz et al., 2020; Walton et al., 2017).

The multigroup analyses further indicated several sex-specific patterns within the dysregulation pathway. Emotional dysregulation was more strongly associated with self-perceived addiction to pornography among men than among women, suggesting that difficulties in emotion regulation may be particularly relevant for how men interpret and label their pornography use as problematic. In addition, lack of premeditation predicted self-perceived addiction to pornography only among men, indicating that impulsive decision-making without consideration of long-term consequences may play a more salient role in men’s self-evaluations of pornography use. Finally, sensation seeking was positively associated with distress among men but not among women, which may reflect gender differences in how novelty-seeking tendencies translate into intensive pornography use and related psychological discomfort. Overall, these findings suggest that the dysregulation pathway may operate differently across sexes, highlighting the importance of considering gender when examining impulsivity, emotional dysregulation, and pornography-related outcomes.

On another note, it is important that all of these findings be interpreted within the theoretical structure of the PPMI model. Although the PPMI framework posits two distinct pathways—the dysregulation pathway and the moral incongruence pathway—these should be conceptualized as separate routes that may coexist but do not necessarily depend on one another. The present work focused exclusively on the dysregulation pathway and, therefore, did not examine interactions or other effects involving moral incongruence. Accordingly, our results highlighted that impulsivity and emotional dysregulation may contribute to distress and self-perceived addiction to pornography independently of morality-related processes. Nevertheless, it should be acknowledged that bidirectional or interactive relationships among emotional dysregulation, moral incongruence, and distress are plausible and should be considered in future research.

In conclusion, we consider that our findings are important contributions to the existing body of literature and enhance the knowledge regarding the PPMI model (Grubbs et al., 2019a) in general, specifically regarding the role of impulsivity and emotional dysregulation in the context of pornography consumption, distress, and self-perceived addiction to pornography. However, the results of the present research must be discussed in relation to both existing limitations and theoretical and practical implications.

### Limitations and Future Directions

Despite the numerous strengths of our research, we must address several limitations. First and foremost, the measurements used in the present work were self-reported. These are inherently subject to a high degree of subjectivity, which may indicate potential for recall bias and social desirability bias among our participants (Adams et al., 1999). This is particularly significant considering that the subject of pornography is taboo in Romania (Gergely & Rusu, 2023; Huţul & Karner-Huţuleac, 2023a, 2024a), and socially desirable responses may arise. At the same time, the study is limited by its cross-sectional nature, which restricts the ability to assess changes over time or establish causal relationships between the studied variables. Cross-sectional studies provide only a snapshot of data at a single point in time, making it difficult to capture temporal dynamics or the progression of the constructs being examined. To gain a more comprehensive understanding and to better account for temporal changes, it is essential for future research to adopt a longitudinal design. Longitudinal studies would enable the tracking of changes in these constructs over time and provide deeper insights into cause-and-effect relationships, thus offering more robust and generalizable findings. The use of path analysis instead of examining the relationships between our variables through a full (measurement and structural) model using Structural Equation Modeling is another limitation of our study. Another limitation of the present work is the high proportion of women in the sample. Thus, although the sample was large, consisting of over 1600 respondents, the predominance of women may pose certain difficulties in generalizing the results. Furthermore, regarding future research directions, it is necessary for our data to be tested in other geographical areas, with different cultural contexts and participant characteristics. Additionally, further research can also focus on the continued development of the PPMI model, testing other individual differences, as well as a potential interaction between these individual differences and aspects related to morality. Another limitation pertains to the use of a single-item measure to assess self-perceived addiction to pornography. While this approach has precedent in the literature (Grubbs et al., 2018c; Huţul & Karner-Huţuleac, 2025; Lewczuk et al., 2020; Petroiu et al., 2025), and captures individuals’ subjective self-labeling, single-item measures may be less reliable than multi-item scales. Future studies should therefore consider using validated multi-item instruments to provide a more nuanced and psychometrically robust assessment of self-perceived pornography-related problems.

Finally, it should be highlighted that the present study was grounded in the development of the PPMI dysregulation pathway and was not intended to test the mediation sequence proposed by the original model. Self-perceived addiction to pornography and distress were therefore examined as parallel outcomes rather than as sequential components of a causal chain. Consequently, future research should explicitly test the complete PPMI model, ideally through longitudinal studies, to examine indirect effects and clarify the temporal ordering among individual differences, self-perceived addiction to pornography, and distress.

### Theoretical and Practical Implications

From a theoretical standpoint, the present research enhances our understanding of the interactions between impulsivity, emotional dysregulation, distress, and self-perceived pornography addiction. The exploration of these variables was conducted within the framework of this study in a Romanian context, which is an understudied one. Thus, this study contributes to the corpus of literature with findings from a different context, that of Eastern Europe, with the majority of existing literature being conducted either in the United States or in Central and Western Europe. Additionally, our findings can serve as a foundation for developing the dysregulation pathway from the PPMI (Grubbs et al., 2019a) model. While other previous works related to the PPMI model have placed primary emphasis on morality-related processes, the present findings suggest that impulsivity and emotional dysregulation may constitute distinct and clinically relevant mechanisms through which pornography use can lead individuals to experience distress and self-perceived addiction. Through the explicit integration of these constructs as individual differences, the present study may contribute to clarifying how pornography-related problems can emerge independently of moral conflict, thereby addressing an important gap in the model’s original formulation. Furthermore, this extension is theoretically important because it allows the PPMI model to more accurately reflect the heterogeneity observed in both clinical and non-clinical populations, in which individuals may experience significant levels of psychological distress related to pornography use despite relatively low moral incongruence. In this sense, the explicit inclusion of these two constructs (impulsivity and emotional dysregulation) within the dysregulation pathway may strengthen the explanatory capacity and clinical relevance of the PPMI framework.

From a practical and clinical perspective, the results of the present study can provide a foundation for personalized interventions for psychotherapists and other mental health professionals who encounter distress related to pornography consumption in their practice. Our findings suggest that practitioners should move beyond explanations that focus exclusively on morality when working with individuals who report significant levels of distress or self-perceived addiction to pornography and should systematically assess impulsivity and emotional dysregulation. Identifying these mechanisms may strengthen an initial starting point for case formulation and guide the planning of therapeutic interventions, particularly by highlighting the need for interventions aimed at fostering optimal emotion regulation strategies, impulse control, and adaptive coping strategies in individuals. Furthermore, in this regard, the results of the present research support the use of transdiagnostic and skills-based therapeutic approaches, such as emotion regulation or impulse management interventions, for individuals experiencing distress related to pornography use. Based on our findings, several psychotherapeutic recommendations were also developed to address distress and self-perceived addiction to pornography (see Table 4).

**Table 4:** Clinical and psychotherapeutic recommendations based on the dysregulation pathway of the PPMI model

No	Focus	Clinical and psychotherapeutic recommendation
1	Assessment beyond morality	Clinicians and psychotherapists should assess impulsivity and emotional dysregulation in clients who present with distress or self-perceived addiction to pornography, rather than assuming that negative outcomes are primarily driven by moral or religious conflicts
2	Screening impulsivity facets	Routine assessment should include multidimensional measures of impulsivity (e.g., lack of premeditation, lack of perseverance, urgency), as different facets may relate to distress and self-perceived addiction to pornography in distinct ways
3	Emotion regulation as a treatment target	Interventions should place strong emphasis on the development of adaptive emotion regulation skills, given the robust associations between emotional dysregulation and distress, as well as with self-perceived addiction to pornography
4	Distinguishing distress from self-perceived addiction to pornography	Clinicians and psychotherapists should distinguish between distress related to pornography use and self-perceived addiction to pornography, given that these outcomes may arise through partially distinct dysregulation mechanisms and may therefore require different therapeutic interventions
5	Addressing lack of premeditation	For clients high in lack of premeditation, therapy should focus on increasing awareness of long-term consequences and strengthening reflective decision-making processes in emotionally charged situations
6	Managing lack of perseverance	Clients exhibiting lack of perseverance may benefit from interventions that enhance frustration tolerance, task persistence, and adaptive coping strategies under stress, reducing pornography as an immediate relief strategy
7	Working with sensation seeking	In clients high in sensation seeking, therapy may focus on helping individuals identify alternative, non-problematic sources of stimulation and novelty, rather than framing pornography use as inherently pathological or suppressing novelty-seeking tendencies
8	Impulse-driven self-labeling	Clinicians and psychotherapists should explore how impulsive tendencies, particularly positive urgency, may contribute to the self-labeling of pornography use as “addictive,” even in the absence of severe distress or functional impairment
9	Personalized case formulation	Case formulation should integrate individual differences in impulsivity and emotional regulation to tailor interventions, rather than relying on a one-size-fits-all explanation of problematic pornography use

## Data Availability

All data associated with the article are available upon request to the corresponding author.
